# Economic evaluation of rotavirus vaccination in children of Bhutan

**DOI:** 10.1016/j.vaccine.2020.05.035

**Published:** 2020-07-06

**Authors:** Pempa, Alia Cynthia G. Luz, Nantasit Luangasanatip, Pritaporn Kingkaew, Deepika Adhikari, Wanrudee Isaranuwatchai, Dechen Choiphel, Clint Pecenka, Frédéric Debellut

**Affiliations:** a Essential Medicines and Technology Division, Department of Medical Services, Ministry of Health, Bhutan; b Health Intervention and Technology Assessment Program (HITAP), Ministry of Public Health (MoPH), Thailand; c Mahidol-Oxford Tropical Medicine Research Unit (MORU), Faculty of Tropical Medicine, Mahidol University, Thailand; d Institute of Health Policy, Management and Evaluation, University of Toronto, Canada; e PATH, USA; f PATH, Switzerland

**Keywords:** Cost-effectiveness analysis, Rotavirus, Vaccine, Bhutan, DALYs, Budget impact analysis, Human resource impact analysis

## Abstract

**Background:**

Diarrhoea remains one of the top ten causes of under-five child morbidity in Bhutan, and rotavirus is a significant cause of child diarrhoeal hospitalisations. This study sought to determine the health outcomes, cost-effectiveness, and budget and human resource implications of introducing rotavirus vaccines in the routine immunisation program to inform Bhutan’s decision-making process.

**Methods:**

We used UNIVAC model (version 1.3.41) to evaluate the cost-effectiveness of a rotavirus vaccination programme compared with no vaccination from a government perspective. We also projected the impact of rotavirus vaccination on human resources and budget. A cost-effectiveness threshold was determined to be 0.5 times the gross domestic product (GDP) per capita (equivalent to the United States dollar ($) 1,537) per Disability-Adjusted Life-Year (DALY) averted. One-way deterministic and probabilistic sensitivity analyses, and threshold analyses were performed to capture parameter uncertainties.

**Results:**

In Bhutan, a rotavirus vaccination programme over 10 years (2020 to 2029) can avert between 104 and 115 DALYs, at an incremental cost ranging from $322,000 to $1,332,000. The incremental cost-effectiveness ratio (ICER) across four vaccination programmes compared to no vaccination scenario were $9,267, $11,606, $3,201, and $2,803 per DALY averted for ROTARIX, RotaTeq, ROTAVAC, and ROTASIIL, respectively. The net five-year budget impact of introducing a rotavirus vaccination programme ranged from $0.20 to $0.81 million. The rotavirus vaccination programme has a potential to reduce the workload of health care workers such as paediatricians, nurses, dieticians, and pharmacists; however, the programme would require an additional 1.93–2.88 full-time equivalent of health assistants.

**Conclusion:**

At the current cost-effectiveness threshold, routine rotavirus vaccination in Bhutan is unlikely to be cost-effective with any of the currently available vaccines. However, routine vaccination with ROTASIIL was under the cost-effectiveness threshold of one times the GDP per capita ($3,074). ROTASIIL and ROTAVAC would provide the best value for money in Bhutan.

## 1 Introduction

Globally, diarrhoea is estimated to account for 8–9% of total under-five (U5) mortality. Rotavirus, the leading cause of severe and fatal diarrhoea in children, accounts for 24–37% of overall U5 diarrhoeal deaths [1], [2], [3]. World Health Organization (WHO)/Centers for Disease Control and Prevention (CDC) estimates 215,000 U5 deaths due to rotavirus in 2013, including 70,109 deaths in southern Asia and a rotavirus positivity rate for the region of 34.1% [3]. The WHO recommends rotavirus vaccines be included in all national immunisation programmes, especially in countries with high diarrhoea mortality rates in children [4].

In Bhutan, the number of diarrhoeal visits to health facilities’ and diarrhoeal mortality have decreased gradually over the last decade due to various public health initiatives. However, diarrhoea remains one of the top ten causes of morbidity in children under five years of age in the country [5], which contributes to increased pressure on the healthcare system. A prospective hospital-based surveillance study in Bhutan showed that one-third of the children aged under five years hospitalised with diarrhoea were infected by rotavirus [6].

Four rotavirus vaccines are currently WHO-prequalified for global use [7]. RotaTeq® (a registered trademark of Merck & Co., Inc.) and ROTARIX® (a registered trademark of GlaxoSmithKline Biologicals SA, used under license by GlaxoSmithKline Inc.) have been prequalified for more than a decade. Two new vaccines, ROTAVAC® (Bharat Biotech International Limited) [8] and ROTASIIL® (Serum Institute of India Pvt. Ltd) [9], were both prequalified in 2018. As of October 2019, 97 countries have introduced rotavirus vaccine, out of which 46 are Gavi-supported countries. An additional 23 countries are planning to introduce rotavirus vaccine, 17 of which are receiving support from Gavi [10].

Health services are publicly funded in Bhutan as stated in the Constitution: “The State shall provide free access to basic public health services in both modern and traditional medicines.” [11]. The Royal Government of Bhutan (RGoB) provides healthcare services to its population for free from primary to tertiary care, including referral care outside the country when necessary. Private practices only occur outside the country. Due to economic growth, Bhutan is transitioning from Gavi support and other donors are reducing their support [11]. With the recent addition of pneumococcal conjugate vaccine, the Expanded Programme on Immunisation (EPI) of Bhutan currently provides 12 vaccines. When introducing any new vaccine, sustainability and affordability are two major concerns for the government. The Bhutan Health Trust Fund (BHTF)—the main funding agency for vaccines and essential drugs in Bhutan—notified the government of the availability of 350 million Ngultrum (US$5 million) for the 2019–2020 fiscal year for all vaccines and essential drugs. This means the government must manage the procurement of all vaccines and essential drugs for a year within that amount ceiling or else pay the remainder out of the government budget.

While rotavirus vaccines have been shown to be cost-effective in low- and middle-income countries (LMICs) [12], [13], [14], [15], [16], [17], [18], [19], evidence of the cost-effectiveness of rotavirus vaccine in Bhutan is still limited. Moreover, vaccine selection is a complex and mufti-factorial decision managed through the Health Technology Assessment (HTA) process in order to inform the policy decision. Prior to the introduction of rotavirus vaccination in the EPI of Bhutan, this study estimates the health outcomes, cost-effectiveness, and budget and human resource implications, of alternative rotavirus vaccine products currently available on the market in order to inform Bhutan’s rotavirus vaccine decision-making.

## 2 Materials and methods

### 2.1 Model

We used the Excel-based UNIVAC model (version 1.3.41), a widely used deterministic static cohort model [20], [21]. This is a single universal vaccine impact and cost-effectiveness decision support model developed at the London School of Hygiene and Tropical Medicine (LSHTM). The model was purposefully designed for use in LMICs to evaluate the impact and cost-effectiveness of various vaccines.

A model-based economic evaluation from a government perspective was performed. We evaluated the impact and cost-effectiveness of a nationwide rotavirus vaccination programme compared with no vaccination scenario (status quo). Four rotavirus vaccines—ROTARIX, RotaTeq, ROTAVAC, and ROTASIIL—were evaluated, assuming administration alongside the Diphtheria, Tetanus, and Pertussis (DTP) vaccine. The model simulated outcomes for children with and without rotavirus vaccine. For rotavirus cases, the model assumes children will experience one of three different health states: no rotavirus gastroenteritis (RVGE), non-severe RVGE, and severe RVGE. For non-severe RVGE cases, patients may either forego treatment or visit the outpatient department (OPD) of a health facility. We assumed that non-severe RVGE cases would recover regardless of health-seeking behaviour, while severe RVGE cases would either recover or die after having either foregone treatment or sought treatment in the OPD, inpatient department (IPD), or OPD then IPD.

Children under five (U5) years of age were the population of interest for the study, and children under one year were the target population for the vaccination programme for all four vaccines. The model followed the costs and health outcomes of 10 cohorts of U5 children starting in 2020 and captured all disease events, interventions, and budget impacts. Health benefits and costs were discounted with a 3% annual rate. All monetary units were converted to the United States (US) dollar ($) ($1 = 72.8 Bhutanese Ngultrum) [22] and adjusted to 2018 values using the consumer price index (CPI) from the National Statistics Bureau of Bhutan [23]. The total number of RVGE cases, visits, hospitalisations, and deaths with and without vaccine were estimated. The quantified outputs included the number of cases and deaths averted, incremental costs due to the vaccination programme, treatment costs averted, and health worker personnel time to treat rotavirus cases (expressed in full time equivalent (FTE) units). Results were presented in terms of the incremental cost-effectiveness ratio (ICER) expressed in US$ per disability-adjusted life-year (DALY) averted.

Although cost-effectiveness thresholds of one to three times the national GDP per capita have been widely used by LMICs in the past, it is now recommended that countries consider contextual factors and establish locally relevant cost-effectiveness ratio thresholds [24]. Based on latest studies and current practice in LMICs and pending the definition of a locally relevant threshold [25], [26], [27], [28], the study team decided to use a threshold of 0.5 times GDP per capita ($ 1,537) for the base case and one times GDP per capita ($ 3,074) for sensitivity analysis [29].

Key stakeholders including clinicians, members of the National Committee for Immunisation Practice (NCIP), representatives from the Community Health Department, Health Management and Information System (HMIS) unit, Royal Center for Disease Control (RCDC), Medical Supplies and Procurement Division (MSPD), Drug Regulatory Authority (DRA), Bhutan Health Trust Fund (BHTF), and EPI managers were involved in the validation process of the conceptual model, input data and modelling outcomes, in accordance with the HTA process guidelines of the Ministry of Health (MoH) [30].

### 2.2 Model input parameters

#### 2.2.1 Disease burden

The diarrhoeal disease burden in Bhutan was estimated using the HMIS database of health records from all health facilities in Bhutan [31], RCDC surveillance data, and a previously published paper on rotavirus diarrhoea in Bhutan [6]. The U5 diarrhoea incidence was derived from OPD and IPD visits adjusted for the rotavirus positivity rate as reported in RCDC surveillance data from the last five years (2014–2018) and Bhutan’s rotavirus study [6]. From the HMIS database, 5% of all diarrhoea visits were hospitalised; therefore, we assumed that non-severe RVGE visit accounted for 95% of the OPD visits and severe RVGE visits accounted for 5% of the OPD visits. A systematic review on the recognition and care-seeking behaviour for childhood illness in developing countries showed that 65% of caregivers sought health care for diarrhoea in Asia [32]. Therefore, we assumed that diarrhoeal visits reported to HMIS represented 65% of the total diarrhoea cases. There was only one diarrhoeal death each in the past three years, which is also laboratory unconfirmed. Because of the relative prevalence of rotavirus compared to other causes of severe diarrhoea, all diarrhoeal deaths reported to HMIS were assumed to be due to rotavirus. To estimate DALYs, the disability weights from the Global Burden of Disease 2013 study [33] were used. The average duration of RVGE illness was seven days for severe cases and three days for non-severe cases [34]. Table 1 shows all input parameters for disease burden. We used local surveillance data to distribute all disease events among weekly age bands from 0 to 260 weeks of age [31].

**Table 1:** Input parameters and sources of data for estimation of disease burden.

Input parameters	Base case	Lower bound	Upper bound	Probability distribution	References
Incidence (per 100,000 under-five children per year)					
Non-severe RVGE cases	3,265	2,588	5,525	Beta-PERT	[32]
Non-severe RVGE visits	2,122	1,682	3,591	Beta-PERT	[31], [6], *RCDC
Severe RVGE cases	582	379	790	Beta-PERT	[32]
Severe RVGE visits	109	88	182	Beta-PERT	**
RVGE Hospitalisations	269	158	332	Beta-PERT	[31], [6], *RCDC
RVGE Deaths	1.7	0	5	Beta-PERT	[31]

Duration of illness (days)					
Non-severe RVGE case	3	3	7	Beta-PERT	[34]
Severe RVGE cases	7	3	7	Beta-PERT	[34]
Disability weights					
Non-severe RVGE case	0.188	0.125	0.264	Beta-PERT	[33]
Severe RVGE cases	0.247	0.164	0.348	Beta-PERT	[33]

Age distribution of disease events					
Age distribution	Cumulative percentage				
< 1 month	1%				*RCDC
< 2 months	6%				
< 3 months	12%				
< 6 months	32%				
< 1 year	53%				
< 2 years	70%				
< 3 years	83%				
< 4 years	90%				
< 5 years	100%				

**RVGE:** Rotavirus Gastroenteritis; **OPD:** outpatient department

* **RCDC:** Royal Center for Disease Control of Bhutan surveillance data;

** **Assumption:** 5% of OPD visits are severe RVGE

#### 2.2.2 Vaccine efficacy and coverage

Rotavirus vaccines, whether two- or three-dose, can be provided alongside DTP vaccination. DTP vaccine coverage in Bhutan was therefore used as a proxy for rotavirus vaccine coverage. As of July 2018, 99% and 98% of the target population in Bhutan received the first dose and the third dose of DTP, respectively [35]. We assumed that the coverage for two-dose and three-dose rotavirus vaccines would be the same. Mothers in Bhutan have been informed by healthcare providers to strictly follow the vaccination schedule as given in the mother and child health (MCH) hand book of Bhutan. Therefore, we assumed that 50% of Bhutanese children would receive their first dose of rotavirus vaccine by eight weeks of age and 99% by 12 weeks of age. Likewise, 50% of children would receive the second dose by 12 weeks of age and 98% of children would receive the second dose by 26 weeks of age. For the third dose of rotavirus vaccine (required for RotaTeq, ROTAVAC, and ROTASIIL only), 50% of children would receive it by 14 weeks and 98% would receive it by 32 weeks.

A *meta*-regression of randomised controlled trials has reported estimates of live oral rotavirus vaccine efficacy by duration of follow-up for different settings characterised by their U5 mortality levels [36]. Being a high-mortality stratum country for U5 children, Bhutan’s rotavirus vaccine efficacy was estimated to be 50% (95% CI: 38–65%) after the first dose, 79% (95% CI: 75–82%) at two weeks after the second or third dose, and 30% (95 CI: 23–57%) at five years after a full course of rotavirus vaccination.

#### 2.2.3 Vaccination programme cost

Bhutan is no longer eligible for Gavi support and therefore does not automatically access the Gavi-negotiated price for rotavirus vaccines [37], [38]. Vaccine prices per dose for ROTAVAC ($0.95) and ROTASIIL ($1.15) were collected from UNICEF and used in the base-case scenario. Since the prices for RotaTeq and ROTARIX were not available from UNICEF during the study period, upper-middle-income countries (UMICs) and LMICs’ average vaccine purchase prices through UNICEF and self-procurement were used for the base case. These prices were taken from the data reported in the WHO vaccine purchase database [39]. Average vaccine prices reported were $3.82 for RotaTeq and $4.22 for ROTARIX. The Gavi price [40] and average non-Gavi price reported to the same database were used as lower and upper bound values, respectively, for sensitivity analysis (see Table 2).

**Table 2:** Input parameters and sources for vaccine programme cost and health care cost.

Input parameters	Base case (median)	Lower bound	Upper bound	Probability distribution	References
**Vaccine price (US$, 2018)**					
ROTARIX	4.22	2.29	8.70	Beta-PERT	LB [39] UB [40]: [39] average of non-Gavi price from MI4A
RotaTeq	3.82	3.50	4.25	Beta-PERT	
ROTAVAC	0.95	0.85	1.15	Beta-PERT	UNICEF [40]
ROTASIIL	1.15	0.95	1.55	Beta-PERT	

**International handling (%)**					
All vaccines	3%	–	–	–	UNICEF [41]

**International delivery (%)**					
ROTARIX	1.13%	–	–	–	Calculation based on UNICEF invoice issued to MoH
RotaTeq	0.67%	–	–	–	
ROTAVAC	0.89%	–	–	–	
ROTASIIL	0.49%	–	–	–	

**Wastage (%)**					
ROTARIX, RotaTeq, and ROTASIIL	5%	–	–	–	[40]
ROTAVAC	30%	–	–	–	

**Incremental health system cost per dose (US$)**					
ROTARIX	1.13	–	–	–	Data collection from VPDP (expenditures in the 1st year only)
RotaTeq	0.76	–	–	–	
ROTAVAC	0.56	–	–	–	
ROTASIIL	0.76	–	–	–	

**Health care cost (US$, 2018)**					
Cost of non-severe and severe RVGE visits	6.82	6.01	7.63	Beta-PERT	Unit costing of outpatient department visit
Cost of severe RVGE hospitalisation	146.83	74.39	238.14	Beta-PERT	Unit costing of inpatient department admission

**LB**: lower bound; **UB**: Upper bound; **RVGE**: Rotavirus Gastroenteritis; **VPDP**: Vaccine Preventable Disease Program; **MoH**: Ministry of Health, Bhutan.

We used UNICEF’s international handling cost standard rate of 3% of the price per dose of the vaccine [41]. We applied wastage rates to each vaccine as reported in Gavi’s detailed product profile [40]. Data from past procurement experience with UNICEF were used to inform the international transportation cost per dose, which was adjusted based on the respective volume of each vaccine. We used information on cold chain volume, dose requirements, and vaccine price to calculate the proportion of international delivery cost per dose for ROTARIX and RotaTeq. However, the cost of negative cold chain storage at central and regional walk-in coolers for ROTAVAC and cost of additional reconstituting steps for ROTASIIL were not factored into the analysis. The number of vaccine doses required was based on the projected population under one year of age in 2020 [42], vaccine coverage, vaccine wastage rate, and the number of scheduled doses of vaccine.

Since the rotavirus vaccination programme would be coupled with other vaccination programmes in the country using the existing pool of transportation facilities and human resources, the EPI manager and MSPD suggested that there would be no additional cost for a new vaccine apart from programme introduction costs (see Table 2). Therefore, in the first year, the incremental health system cost per dose of ROTARIX, RotaTeq, ROTAVAC, and ROTASIIL was $1.13, $0.76, $0.56, and $0.76, respectively. This includes costs of information, education, and communication materials as well as health worker training.

#### 2.2.4 Health care costs

The unit costs of OPD visits and IPD admissions were estimated from an updated 2009/2010 unit costing of health care services in Bhutan [43]. The update covered capital investments in hospitals, outreach clinics, and high-cost medical equipment (CT scan machines, MRI machines, X-ray machines, and analysers) from 2009 to 2018. All costs were adjusted to 2018 using consumer price index (CPI) from the National Statistics Bureau of Bhutan and equivalent annualised costs were used to calculate the health care costs. The estimated lifetime period (70 years for buildings and 3–10 years for medical equipment) and the depreciation rate for hospital buildings (3.5%) were informed by the 2016 Property Management Manual of Bhutan. Expenditures incurred by hospitals during the 2017/2018 fiscal year on salary and allowances, pension and provident fund contributions, benefits for staffs, and hospital utility bills for electricity, water, and sewage were also included. A total of 37 health workers (from 13 health facilities representing all levels of health care facilities in Bhutan) involved in treating diarrhoea patients were interviewed using cost collection forms to estimate the cost of service delivery. Integrated Management of Neonatal and Childhood Illnesses (IMNCI) guidelines from the Ministry of Health [44] were used to cross-verify the treatment strategy for diarrhoea. The information on prices of drugs and consumables were from MSPD.

We estimated that the unit cost of treatment for non-severe and severe RVGE at OPD was $6.82 ($6.01 - $7.63) and the unit cost of treatment for hospitalised severe RVGE was $146.83 ($74.39 - $238.14) (see Table 2).

### 2.3 Incremental cost-effectiveness ratio

The cost-effectiveness results were presented in terms of incremental cost-effectiveness ratios (ICERs) expressed as the cost per DALY averted. In the absence of an existing country threshold for interpreting cost-effectiveness results in Bhutan, the study team chose a cost-effectiveness threshold of 0.5 times the GDP per capita, acknowledging the evolving norms of cost-effectiveness threshold use [26]. One times the GDP per capita was also used as an alternative threshold, reflecting previous practice in Bhutan [45].

### 2.4 Uncertainty analyses

A series of uncertainty analyses (i.e., scenario analysis, deterministic and probabilistic sensitivity analysis or PSA, and threshold analysis) were performed to capture uncertainty across various data inputs. For sensitivity analysis, we used lower and upper bound values of a series of input parameters, focusing on disease event incidence rates, vaccine prices, and health care costs. For scenario analyses, eight different scenarios were performed using the lower and upper bound of input parameters given in Table 1, Table 2: (1) low disease burden; i.e., lower bound of the incidence input parameter (2) high disease burden; i.e., upper bound of the incidence input parameter; (3) low vaccine price; i.e., lower bound of vaccine price; (4) high vaccine price; i.e., upper bound of the vaccine price; (5) low health care costs; i.e., lower bound of cost input parameter; (6) high health care costs; i.e., upper bound of the cost input parameter; (7) most favourable scenario, i.e., lower price of the vaccine, high disease burden, and high health care cost; and (8) least favourable scenario; i.e., higher price of the vaccine, low disease burden, and low health care cost. A PSA was performed with 1,000 iterations of Monte-Carlo simulation to yield a range of possible values for costs and outcomes. A cost-effectiveness acceptability curve (CEAC) was built using net monetary benefit values and cost-effectiveness results. A threshold analysis was performed to determine the optimum price of the vaccines from a given set of different cost-effectiveness thresholds.

### 2.5 Budget impact analysis

Budget impact analysis was performed to estimate the rotavirus vaccination programme costs and healthcare costs over five years of vaccination programme implementation compared with a no vaccine scenario. All costs were undiscounted and the budget represents the monetary value in 2018.

### 2.6 Impact on human resources for health

A separate model for human resource (HR) impact (i.e., quantity, task, and productivity or QTP) was constructed [46]. The impact was expressed in terms of full-time equivalent (FTE), which is equal to one employee working on a full-time basis for one year. Health professionals including 3 paediatricians, 10 general doctors, 4 health assistants, 10 nurses, and 10 pharmacists/pharmacy technicians from 13 health facilities representing all levels of health care facilities in Bhutan were interviewed to estimate the amount of time required to treat a diarrhoea patient, from consultation to dispensing drugs, and to vaccinate a child with a full course of rotavirus immunisation (using a two- or three-dose vaccine). FTE for each category of health workers for treatment of diarrhoea and vaccination of children was determined. The total FTE was adjusted for public holidays, annual leave, and numbers of working hours [47], [48]. The FTE of health workers is the product of net working days per year and working time (in minutes) per day. One FTE corresponded to 83,760 min per health worker per year. FTE was calculated using the formula below.

$$\mathrm{FTE}=\frac{\mathrm{Total}\;time\;spent\;by\;healthcare\;provider\;per\;case*Number\;of\;cases\;or\;target\;population}{\mathrm{Total}\;working\;time\;per\;year}$$

## 3 Results

### 3.1 Health benefits, economic impacts and cost-effectiveness

Without a rotavirus vaccination programme, 248 DALYs were estimated to be lost due to illness related to diarrhoea (25,336 RVGE cases) and deaths (8 deaths) over a ten-year period (2020 to 2029). Also, without the vaccination programme, there would be 14,692 RVGE visits and 1,772 RVGE hospitalisations, which would cost approximately $313,000. To implement a rotavirus vaccination programme for 10 years, the cost of the vaccination programme would be $1.09 million, $1.48 million, $0.51 million, and $0.48 million for ROTARIX, RotaTeq, ROTAVAC, and ROTASIIL, respectively. The vaccination programme would prevent 3 to 4 deaths, avert 104 to 115 DALYs, and reduce healthcare costs by $131,000 to $145,000 over a decade of programme implementation. Table 3 shows discounted health benefits, economic impacts, and incremental cost-effectiveness results of each rotavirus vaccine for 10 birth cohorts.

**Table 3:** Health benefits, economic impacts, and incremental cost-effectiveness results of rotavirus vaccines (10 birth cohorts, discounted at 3% per annum).

Health benefits and economic impact	ROTARIX (2 doses)	RotaTeq (3 doses)	ROTAVAC (3 doses)	ROTASIIL (3 doses)
Health benefits (number averted), base case (95% confidence interval)				
RVGE cases averted (severe + non-severe)	10,573 (8,729–15,441)	11,636 (9,680–16,846)	11,636 (9,680–16,846)	11,636 (9,683–16,846)
RVGE visits averted (severe + non-severe)	6,130 (5,106–9,096)	6,747 (5,665–9,924)	6,747 (5,665–9,924)	6,747 (5,673–9,924)
RVGE hospitalisations averted	739 (561–937)	814 (621–1,019)	814 (621–1,019)	814 (621–1,019)
Deaths averted	3.32 (1–8)	3.66 (2–8)	3.66 (2–8)	3.66 (2–8)
DALYs averted	104 (54–223)	115 (59–245)	115 (59–245)	115 (59–245)

Economic impact (US$), base case (95% confidence interval)				
Vaccine programme cost	1,098,000 (757,948–1,771,052)	1,477,000 (1,286,391–1,677,021)	512,000 (444,363–603,901)	467,000 (395,308–578,138)
Healthcare cost averted	131,000 (91,612–189, 964)	145,000 (101,331–206,754)	145,000 (101,331–206,754)	145,000 (101,331–206,754)
Incremental cost	967,000	1,332,000	367,000	322,000

Incremental cost-effectiveness ratio (ICER) in US$ per DALYs averted compared to no vaccine scenario, base case (95% confidence interval)				
ICER	9,267 (3,738–22,393)	11,606 (5,325–22,949)	3,201 (1,312–6,941)	2,803 (1,135–6,201)

**RVGE**: Rotavirus Gastroenteritis; **DALY**: Disability-adjusted life-year; **ICER**: Incremental cost-effectiveness ratio.

The incremental cost-effectiveness ratios (ICER) of the four vaccination programmes compared to no vaccination scenario were $9,267, $11,606, $3,201 and $2,803 per DALY averted for Rotarix, Rotateq, ROTAVAC, and ROTASIIL, respectively. None of the vaccines appears to be cost-effective at a threshold of 0.5 times the GDP per capita ($1,537) from the government perspective. The ICER for ROTASIIL falls just below the one times the GDP per capita threshold ($3,074), and the ICER for ROTAVAC slightly exceeds the one times the GDP per capita threshold.

### 3.2 Budget impact

A five-year budget impact analysis revealed that the vaccination programme is expected to reduce treatment cost by 42% to 46%. However, the net budget impact would be $0.59 million, $0.81 million, $0.23 million, and $0.20 million for ROTARIX, RotaTeq, ROTAVAC, and ROTASIIL, respectively, as shown in Table 4.

**Table 4:** Budget impact analysis for the first five years of the introduction of vaccination programme (undiscounted costs, US$).

Year	No Vaccine	ROTARIX	RotaTeq	ROTAVAC	ROTASIIL
*Vaccine programme costs*					
**Year 1**	**–**	163,000	210,000	84,000	86,000
**Year 2**	**–**	130,000	176,000	60,000	53,000
**Year 3**	**–**	127,000	173,000	58,000	52,000
**Year 4**	**–**	124,000	168,000	57,000	50,000
**Year 5**	**–**	121,000	163,000	55,000	49,000
Total	–	**665,000**	**890,000**	**314,000**	**289,000**

*Health care costs*					
**Year 1**	38,000	22,000	21,000	21,000	21,000
**Year 2**	38,000	22,000	20,000	20,000	20,000
**Year 3**	37,000	22,000	20,000	20,000	20,000
**Year 4**	37,000	21,000	20,000	20,000	20,000
**Year 5**	36,000	21,000	20,000	20,000	20,000
Total	**187,000**	**109,000**	**101,000**	**101,000**	**101,000**

*Net budget impact*					
**Year 1**	**–**	147,000	192,000	66,000	68,000
**Year 2**	**–**	114,000	159,000	42,000	36,000
**Year 3**	**–**	112,000	156,000	41,000	34,000
**Year 4**	**–**	109,000	151,000	40,000	33,000
**Year 5**	**–**	105,000	147,000	39,000	32,000
Total	–	**587,000**	**805,000**	**228,000**	**203,000**

**Note:** Figures rounded to the nearest thousand US$.

### 3.3 Human resource impact

The rotavirus vaccination programme would require an additional 1.93–2.88 FTE of health assistants, who would be involved in vaccinating children, while FTE of other health workers like doctors, nurses, dieticians, and pharmacists would be reduced (ranging from 0.01 to 0.16) (see Table 5).

**Table 5:** Human resource impact analysis by type of health professional (full time equivalent in one year).

Health personnel	Human resource requirement (FTE)			Displaced human resource (FTE)	
	Vaccination (ROTARIX)	Vaccination (Others)	No vaccination (treatment)	ROTARIX	Others
Dietician	–	–	0.02	− 0.01	− 0.01
General doctor	–	–	0.35	− 0.04	− 0.16
Nurse	–	–	0.09	− 0.04	− 0.04
Health assistants	2.01	2.98	0.25	+ 1.93	+ 2.88
Paediatrician	–	–	0.35	− 0.04	− 0.16
Pharmacist	–	–	0.06	− 0.02	− 0.03

**HR**: Human resource; **FTE:** full time equivalent.

### 3.4 Scenario analysis

**Fig. 1:**
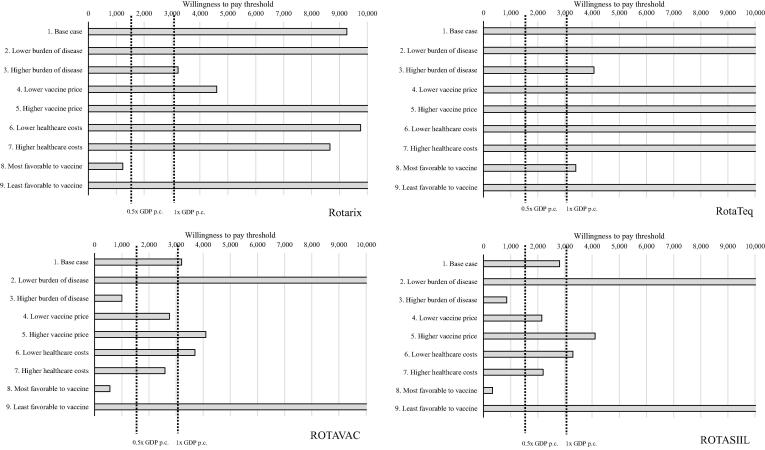
Scenario analysis results, showing incremental cost-effectiveness ratio of ROTARIX, RotaTeq, ROTAVAC, and ROTASIIL compared with no vaccination (reported in US$ per DALYs averted). Legend: GDP p.c.: gross domestic product per capita.

**Fig. 2:**
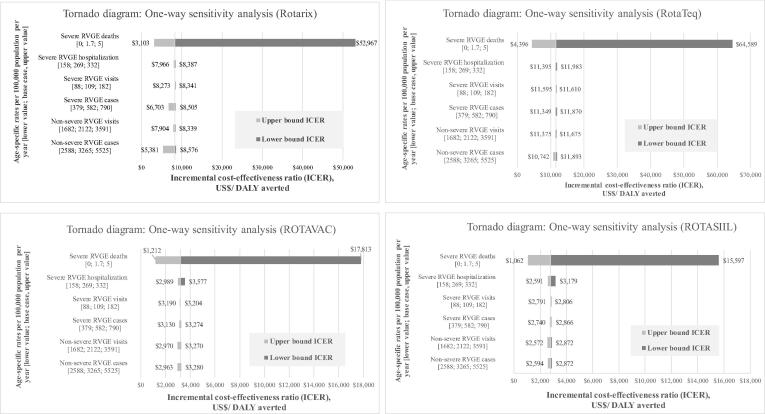
Tornado diagram showing results of one-way sensitivity analyses of disease burden parameters. Legend: RVGE: rotavirus gastroenteritis; ICER: incremental cost-effectiveness ratio.

From the eight scenarios (see Fig. 1), at a threshold of 0.5 GDP per capita, ROTARIX is cost-effective only in the most favourable scenario; i.e., when the price of vaccine is low ($2.29), the disease burden is high (upper bound of the incidence input parameter), and the healthcare costs for diarrhoea treatments are high ($7.63 for OPD visit and $238.14 for hospitalisation). None of the scenarios exploring RotaTeq are cost-effective at that threshold. Conversely, ROTAVAC and ROTASIIL are cost-effective at a threshold of 0.5 times the GDP per capita when accounting for higher disease burden (i.e., upper bound of the incidence input parameter). One-way sensitivity analysis shows that the mortality rate is the most sensitive parameter affecting the results (see Fig. 2).

**Fig. 3:**
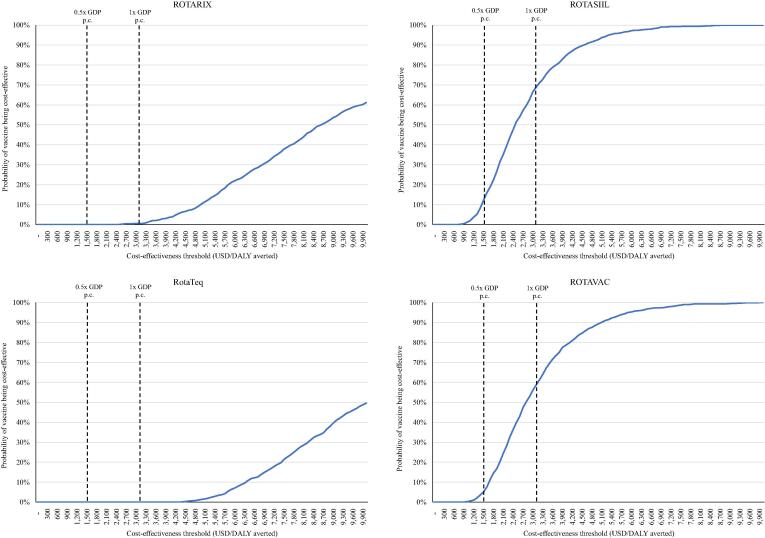
Cost-effectiveness acceptability curve for the probabilistic sensitivity analysis of ROTARIX, RotaTeq, ROTAVAC, and ROTASIIL. Legend: GDP p.c.: gross domestic product per capita; DALY: Disability-adjusted life-year.

At a threshold of one times the GDP per capita, ROTAVAC and ROTASIIL are both likely to be cost-effective in the scenarios considering a higher burden of disease, lower vaccine prices (Gavi-negotiated price), and higher healthcare costs. Fig. 3 shows a cost-effectiveness acceptability curve for each rotavirus vaccine, summarising results from the probabilistic sensitivity analysis. It shows that at a cost-effective threshold of 0.5 times the GDP per capita, ROTASIIL has the highest chance (12%) of being cost-effective followed by ROTAVAC (5%), ROTARIX (0%), and RotaTeq (0%). At one times the GDP per capita, ROTASIIL has the highest chance (69%) of being cost-effective followed by ROTAVAC (59%), ROTARIX (1%), and RotaTeq (0%). ROTARIX and RotaTeq would be cost-effective at 0.5 times the GDP per capita if their respective vaccine prices per dose are not more than $1.02 and $0.75, respectively. Similarly, ROTAVAC and ROTASIIL would be cost-effective at 0.5 times the GDP per capita if their vaccine prices per dose were $0.57 and $0.76 or lower, respectively (see Table 6).

**Table 6:** Threshold analysis on rotavirus vaccine price.

	ROTARIX	RotaTeq	ROTAVAC	ROTASIIL
ICER, compared to no vaccination scenario (US$ per DALYs averted)	9,267	11,606	3,201	2,803
Vaccine price in the model (US$ per dose)	4.22	3.82	0.95	1.15

**Vaccine price (US$ per dose) being cost-effective at**				
0.5 GDP per capita	1.02	0.75	0.57	0.76
1 GDP per capita	1.65	1.22	0.92	Cost-effective at the current price

**DALY:** Disability-adjusted life-year; **ICER:** incremental cost-effectiveness ratio; **GDP:** gross domestic product.

## 4 Discussion

Without Gavi support and compared to a “no vaccine” scenario, the introduction of rotavirus vaccines into the routine immunisation programme in Bhutan would not be cost-effective at the threshold of 0.5 times the GDP per capita. Among the four vaccines, ROTASIIL has the best value for money with its lower price and budgetary impact while maintaining comparable health outcomes. ROTAVAC, ROTARIX, and RotaTeq are, respectively, the second, third, and least cost-effective vaccines.

Aside from cost-effectiveness, financial sustainability is a key consideration for Bhutanese decision makers who are contending with their transition from Gavi support. Expected expenditure, budgets, and priorities would be important inputs for the decision on whether to invest in the rotavirus vaccine. For instance, one important assumption taken is the coupling of the rotavirus vaccination programme with the country’s other vaccination programmes. The programme will be using the existing pool of transportation facilities and human resources, thereby resulting in no additional cost apart from the programme introduction and incremental health system cost per dose. To introduce ROTASIIL in the routine immunisation, the government would need to commit an amount of $289,000 million for five years. This may require additional monitoring and programme evaluation of the actual costs. Further, the Royal Government of Bhutan can use the results from this study with the considerations of different stakeholders to inform resource allocation. The budget impact per year for rotavirus vaccination is around 1.2% of the total amount ($5 million) that the BHTF provides annually to the MOH.

A rotavirus vaccination programme has the potential to reduce the workload of doctors, nurses, dieticians, and pharmacists involved in treating diarrhoea, but the vaccination programme would result in an increased workload for health assistants. Considering there are only around 10 paediatricians currently available in the country, reducing the workload of paediatricians (0.35 FTE) would be significant. However, the introduction of the vaccine would increase the workload of the vaccination programme managers and providers, such as health assistants, by around two to three FTE.

The study findings are similar to those in France, Ireland, the Netherlands, and Spain [49], [50], [51], [52]. This study also used the same analytical model – the UNIVAC – as those done in Afghanistan, South Korea, and Mongolia [12], [16], [53]. The UNIVAC model has proven to be a useful tool for building capacity to conduct HTA research and the vaccine prioritisation process in settings where there is an HTA research capacity constraint. Similar to the previous studies, the cost-effectiveness result of our study was most sensitive to disease incidence and vaccine price. The rotavirus disease burden—especially RVGE deaths—and vaccine prices are found to be the primary drivers of costs. If Bhutan had a higher rotavirus diarrhoea burden than currently estimated, high treatment costs, and access to lower vaccine costs at Gavi prices (i.e., the price of vaccines meant for countries currently under Gavi support), ROTAVAC, ROTASIIL, and ROTARIX would be cost-effective. The price per dose of ROTARIX, RotaTeq, ROTAVAC, and ROTASIIL would need to undergo price negotiation and decrease by an estimated 76%, 80%, 40%, and 34%, respectively, from their current price estimates. However, price negotiation strategies to reduce vaccine prices may be inapplicable given Bhutan’s small birth cohort (12,000 births per year) compared to countries with a larger target population such as Thailand [54]. Pooled vaccine procurement with other nations in the region [55] to collectively improve negotiating power may be suitable in the Bhutanese context.

Bhutan does not have a predetermined cost-effectiveness threshold. Although the one times GDP per capita was previously used as a threshold to determine the value for money of pneumococcal conjugate vaccine in Bhutan [45], 0.5 times the GDP per capita was used as the base case in this study and the one times GDP per capita was used in the sensitivity analysis. The use of a lower threshold is based on findings from recent studies suggesting that thresholds representing health opportunity costs in LMICs tend to be lower than one times the GDP per capita [25], [26]. The study team recognised the evolving nature of the cost-effectiveness thresholds discussion and decided to use a more restrictive threshold. While more stringent, this threshold was not the result of a rigorous analysis of the current opportunity costs of health expenditure in Bhutan. The implications of our results would be more favourable to rotavirus vaccination, had a somewhat less stringent threshold been selected. Having a local country-specific cost-effectiveness threshold that is more grounded in local data would be useful in future economic studies in Bhutan.

This study acknowledges several limitations. First, there was uncertainty around vaccine prices and vaccine management costs. Confirming such prices with the manufacturers, particularly for ROTARIX and RotaTeq, is a critical step that the study team was not able to achieve during the study. Additionally, neither the requirement of negative cold chain storage at central and regional walk-in coolers for ROTAVAC nor the additional steps for reconstitution of ROTASIIL were factored into this analysis. These costs should be considered for a nationwide vaccine introduction due to more training and time needed for vaccine administration. Second, although vaccine wastage rate depends on each country’s health care setting and management, we conservatively assumed a 30% wastage rate for ROTAVAC because of its five-dose presentation. With somewhat similar ICER values for ROTAVAC and ROTASIIL, one can assume that a lower wastage rate associated with the use of ROTAVAC in Bhutan would probably yield equivalent results for both vaccines. Third, there is a high probability of under-reporting of rotavirus diarrhoea cases owing to the limited diarrhoea surveillance and sentinel sites in Bhutan. Moreover, the number of stool samples collected is low, which could have led to a low rotavirus positivity rate. To account for the potential underestimation of the RVGE burden, we used previously published rotavirus positivity values, based on more robust data, as the upper bound for calculating the disease burden incidence (used in our scenario 2) [6]. Fourth, we assumed there was only one diarrhoeal death per year reported in Bhutan in the past few years. We assumed the death was due to rotavirus despite a lack of laboratory confirmation. Therefore, we could have overestimated the rate of RVGE deaths. Fifth, costs related to the increase in human resources or FTE requirements for vaccination were excluded, instead assuming additional tasks would be manageable with existing human resources. Sixth, opportunity costs and indirect health benefits of rotavirus vaccination, such as the reduction of febrile seizure amongst vaccinated children as well as the likelihood of an indirect protective effect on the unvaccinated population (herd immunity), were omitted. Despite this, evidence of such circumstances exist in a number of settings [56], [57], [58]. With this current approach, our estimated ICERs are conservative and undervalue the benefits of the vaccination programme. Seventh, the lack of local data on the background risk of intussusception in Bhutan limited the exploration of the potential for an increased number of cases of intussusception as an adverse event of rotavirus vaccination. However, the risk of intussusception death is likely to be insignificant due to the small birth cohort and accessible health care for intussusception in Bhutan. Moreover, recent analysis reconfirms the favourable benefit-risk profile (rotavirus gastroenteritis deaths prevented per excess intussusception death) of rotavirus vaccines in LMICs [59]. Lastly, some of the parameter values used in the model were derived from the literature. This includes the care-seeking behaviour used to estimate the baseline burden of rotavirus infection and treatment duration amongst RVGE cases [32], [34]. If the values of these parameters are lower than the actual data, the overall baseline burden of rotavirus infection would be higher. In such a scenario, the vaccine could become cost-effective due to additional savings from the treatment costs and provide more health benefits in terms of DALYs averted from the prevention of more rotavirus infection cases.

In conclusion, none of the rotavirus vaccines evaluated in this study is likely to be cost-effective for routine immunisation in Bhutan at a threshold of 0.5 times the GPD per capita. However, of the four rotavirus vaccines assessed, ROTAVAC and ROTASIIL have better value for money and could potentially be cost-effective in Bhutan if vaccine prices were lower, the rotavirus burden was higher than the current estimate, or if a different cost-effectiveness threshold was used.

## CRediT authorship contribution statement

**Pempa:** Conceptualization, Formal analysis, Investigation, Writing - original draft. **Alia Cynthia G. Luz:** Conceptualization, Investigation, Project administration, Writing - review & editing. **Nantasit Luangasanatip:** Conceptualization, Formal analysis, Investigation, Methodology, Writing - review & editing. **Pritaporn Kingkaew:** Conceptualization, Formal analysis, Investigation, Methodology, Writing - review & editing. **Deepika Adhikari:** Investigation, Project administration, Writing - review & editing. **Wanrudee Isaranuwatchai:** Conceptualization, Supervision, Writing - review & editing. **Dechen Choiphel:** Investigation, Writing - review & editing. **Clint Pecenka:** Writing - review & editing. **Frédéric Debellut:** Conceptualization, Formal analysis, Investigation, Methodology, Writing - review & editing.

## Declaration of Competing Interest

The authors declare that they have no known competing financial interests or personal relationships that could have appeared to influence the work reported in this paper.
